# Incidence and Related Factors for Hospital-Acquired Pneumonia Among Older Bedridden Patients in China: A Hospital-Based Multicenter Registry Data Based Study

**DOI:** 10.3389/fpubh.2019.00221

**Published:** 2019-08-13

**Authors:** Jing Jiao, Xiang-yun Yang, Zhen Li, Yan-wei Zhao, Jing Cao, Fang-fang Li, Ying Liu, Ge Liu, Bao-yun Song, Jing-fen Jin, Yi-lan Liu, Xian-xiu Wen, Shou-zhen Cheng, Lin-lin Yang, Xin-juan Wu, Jing Sun

**Affiliations:** ^1^Chinese Academy of Medical Sciences, Peking Union Medical College, Peking Union Medical College Hospital, Beijing, China; ^2^Beijing Key Laboratory of Mental Disorders, The National Clinical Research Center for Mental Disorders, Beijing Anding Hospital, Capital Medical University, Beijing, China; ^3^Henan Provincial People's Hospital, Zhengzhou, China; ^4^The Second Affiliated Hospital, Zhejiang University School of Medicine, Hangzhou, China; ^5^Wuhan Union Hospital, Wuhan, China; ^6^Sichuan Provincial People's Hospital, Chengdu, China; ^7^The First Affiliated Hospital, Sun Yat-sen University, Guangzhou, China; ^8^School of Nursing, Qingdao University, Qingdao, China; ^9^School of Medicine, Griffith University, Gold Coast, QLD, Australia

**Keywords:** hospital-acquired pneumonia, older bedridden patients, incidence density, related factors, registry data-based study

## Abstract

**Objective:** To identify the incidence and related factors for hospital-acquired pneumonia (HAP) among older bedridden patients in China.

**Study design and setting:** This multicenter registry data-based study conducted between November 2015 and March 2016 surveyed 7,324 older bedridden patients from 25 hospitals in China (six tertiary, 12 non-tertiary, and seven community hospitals). The occurrence of HAP among all participants was monitored by trained investigators. Demographics, hospitalization information and comorbidity differences were compared between patients with and without HAP. A multilevel regression analysis was used to explore the factors associated with HAP.

**Results:** Among 7,324 older bedridden patients, 566 patients were diagnosed with HAP. The incidence of HAP in this study was 13.9 per 1,000 person-days. There were statistical differences in gender, age, length of bedridden days, BMI, smoking, department, undergoing general anesthesia surgery, ventilator application, Charlson comorbity index (CCI) score, disturbance of consciousness, tranquilizer use, glucocorticosteroid use, and antibiotic use between patients with HAP and patients without HAP (all *p* < 0.05). Multilevel regression analysis found no significant variance for HAP at the hospital level (0.332, *t* = 1.875, *p* > 0.05). There were significant differences for the occurrence of HAP among different departments (0.553, *t* = 4.320, *p* < 0.01). The incidence density of HAP was highest in the ICU (30.1‰) among the selected departments, followed by the departments of neurosurgery (18.7‰) and neurology medicine (16.6‰). Individual patient-level factors, including older age, disturbance of consciousness, total CCI score, ICU admission, and glucocorticoid and antibiotic use, were found to be associated with the occurrence of HAP (all *p* < 0.05).

**Conclusion:** A relatively high incidence density of HAP among older bedridden patients was identified, as well as several factors associated with HAP among the population. This suggests that attention should be paid to the effective management of these related factors of older bedridden patients to reduce the occurrence of HAP.

## Introduction

The number of bedridden patients increases with the increasing prevalence and incidence of diseases among older population (1). Staying in bed at hospital could cause many complications, and pneumonia is one of the most common complications. Hospital-acquired Pneumonia (HAP) is a major nosocomial infection worldwide resulting in increased morbidity, mortality, and medical costs. Older bedridden patients, whose basic physiological needs are carried out in bed, often accompanied by worse immune, swallowing and respiratory function, are at high risk of HAP (2). Approximately 1.1–1.5% of all hospital patients developed HAP (3), and 5.8–8.3% of older hospitalized patients developed HAP in western countries (2, 4). In China, the incidence of hospital- acquired infections in all hospitalized patients was 3.22–5.22%, of which hospital acquired lower respiratory tract infections were 1.76–11.94%. Patients over 65 years old are the main group of HAP, accounting for about 70% (5). The incidence of and related factors of HAP among older bedridden patients is still not clear in China.

Identifying the factors associated with HAP is important to prevent HAP and reduce the incidence of HAP. This may ultimately decrease length of hospital stay, mortality, reduce inappropriate antibiotic use, and to improve functional outcomes. Several studies have identified age, smoking, chronic pulmonary disease, type of surgery, malnutrition, state of unconsciousness during hospitalization, mechanical ventilation, use of a nasogastric tube, multi-trauma and poor health condition are potential risk factors for the HAP (6–8). These studies mainly investigated the risk factors for ventilator- associated pneumonia in intensive care units (ICUs). Several studies have investigated the incidence of HAP in non-ICU patients. They found the incidence of HAP outside the ICU was 2–4 cases per 1,000 patients. The risk factors associated with HAP in non-ICU patients include age, malnutrition, steroid use, chronic renal failure, anemia, unconscious, comorbidity, recent hospitalization, and thoracic surgery (9–11). These findings suggest that the risk factors for HAP vary among patients in different wards. Therefore, it is important to understand the occurrence of and factors associated with HAP in various departments for the prevention of nosocomial infections.

In hospitals, nurses are responsible for most of the life care, nursing and treatment of bedridden patients. Identifying the risk factors of HAP is important for improving the quality of nursing and therapeutic effect of bedridden patients. However, our previous study surveyed the nurses' knowledge and attitudes on complications of bedridden patients including HAP, and found their knowledge on the related factors are not adequate (12). Therefore, it is necessary to understand the incidence of HAP and related factors among bedridden patients in China. To our best knowledge, no study has estimated the incidence of HAP among older hospitalized bedridden patients in general wards. It is not clear which factors contribute to bedridden HAP in China. To fill this research gap, this study aimed to identify the prevalence and related factors of HAP among older bedridden patients in various departments (ICU, orthopedics, neurology medicine, neurosurgery, general surgery, cardiovascular, other surgery and other medicine) in China by using a nationwide multicenter hospital-based study sample during November 2015 to March 2016.

## Methods

### Study Design, Setting, and Population

**Figure 1:**
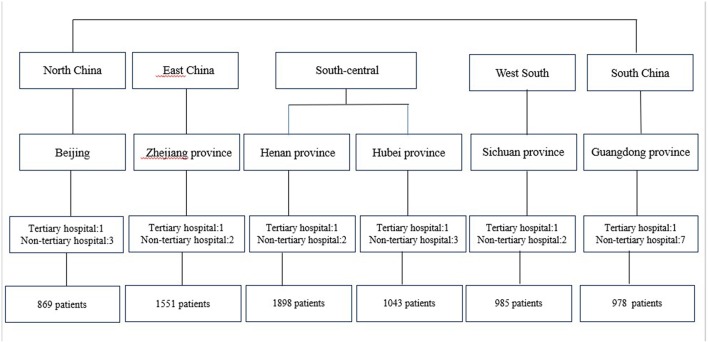
Sampling recruitment procedure.

This multicenter study was designed to assess the incidence of HAP in participants during hospitalization. The data used in the current study is a part of a national research program that aimed to construct a standardized nursing intervention model for major complications of immobility (MCI) among bedridden patients. This study adopted a multi-level and purposive sampling method which is a convenient in nature but has clear inclusion criteria to achieve the purpose of the representation of the sample. All departments were embedded within hospitals, and hospitals were within provinces. To meet the inclusion criteria at all level's data, first, six provinces or municipality cities were selected including Beijing, Henan province, Hubei province, Zhejiang province, Guangdong province and Sichuan province to represent the North, South, West, East and Central parts of China. Second, one of the largest tertiary hospital with over 2,000 beds in each province and at least two non-tertiary hospitals in each province, with total 25 hospitals that met the inclusion criteria, were recruited. Third, the department in which older bedridden patients accounted for more than 20% of the total number of patients was selected from each hospital, including ICU, neurology medicine and other medicine, neurosurgery, general surgery and other surgery, orthopedics and cardiovascular departments. From November 1, 2015 to March 17, 2016, participants who met the inclusion criteria were continuously recruited into the study (specific sampling methods see Figure 1). Bedridden Patients were defined as all patients whose basic physiological needs were carried out in bed because of their illness or weakness except active or passive bedside standing/wheelchair use when examination or treatment was conducted. Patients were recruited if they (1) were 65 years old and over; (2) had stayed in bed for at least 24 h when he/she was surveyed; (3) understood the aims of the study and signed the consent form. Patients were excluded from data analysis if they had community-acquired pneumonia and more than one type of MCI at the time of enrollment.

### Variables and Data Collection

HAP refers to pulmonary parenchymal infection caused by pathogenic bacteria existing in hospital environment, which occurs 48 h after the patient's admission. Ventilator- associated pneumonia (VAP) was included in this study based on the practice guideline for HAP/VAP in China. HAP was diagnosed based on a combination of clinical, laboratory and radiological data by physicians and were recorded on the medical records (5). Patients' socio-demographic characteristics were collected using a questionnaire. To ensure detailed information for age, education and BMI was analyzed, age was categorized into five groups, namely a 65–69 years old group, a 70–74 years old group, a 75–80 years old group, a 80–85 years old group, and a ≥85 years old group. The education level of patients was divided into illiteracy, primary school, middle school, senior high school, college and above. The grouping of age and education level was based on the distribution of the participants, which ensures that each group has enough sample size to increase the balance between groups. BMI was then used to divide them into 18.5–23.9, <18.5, >23.9 and <28, and <28 kg/m^2^ groups according to the guidelines for prevention and control of overweight and obesity in Chinese adults (13). The smoking status of patients was classified as never smoked, smoking and ex-smokers. Medical comorbidities assessment and hospitalization information use were collected from the audits of the hospital chart and electronic health records. The Charlson comorbidity index (CCI) score was calculated as an important measure of baseline comorbid conditions of the patients. All patients' treatments during hospitalization, including general anesthesia surgery, ventilator use, tranquilizer use, antibiotic use, and glucocorticoid use, were investigated using yes or no questions. The number of bedridden days, hospitalization time, and days from admission to diagnosis of HAP, and other related medical information of all patients, were extracted from medical records and recorded. A case report form and an Electronic Data Collection system (EDC) were designed for the data collection work for this study.

The trained nurses collected data of all participants from admission to discharge. To guarantee the quality of the data collection for the study, a project manual was developed. Two registered nurses in each department were trained by the project team members were responsible for bedridden patient data collection, including recording the patient bedridden status and diagnosis of pneumonia on a daily basis. The head nurses of each department were responsible for information checking and review. In addition, a project team regularly conduct on-site quality control and case verification and provide feedback via email, phone, and meeting.

### Ethical Considerations

This study was approved by the Ethical Committee of Peking Union Medical College Hospital (S-700). Patients received verbal and written information about the study and provided written consent to participate. If patients had a cognitive impairment (e.g., because of dementia), their contact person was asked to provide the consent. Patients were advised they have right to withdraw from the study at any time, and the care they received would not be affected by their participation status. All data were kept confidential and processed anonymously.

### Data Analysis

First, we explored data distribution. Continuous variables were examined for the normality. Then the normal data was presented as mean and standard deviation (SD), non-normal data was presented as median and interquartile range (IQR). Dichotomous variables were described as frequency and percentage (14). Second, in the univariate analysis, demographics, hospitalization information, and comorbidity differences between patients with and without HAP groups were assessed using the Student's *t*-test for continuous variables and the Chi-squire test for categorical variables. Considering the lengths of stay in hospital may affect the occurrence of HAP, we calculated the incidence density of HAP using the total number of person-days as denominator. All statistical tests were performed at an alpha level of 0.05. Two-tailed estimates of effect and 95% confidence interval were reported for all regression coefficients. SPSS version 24 was used to analyze the data.

Further, we analyzed the association between HAP and the variables determined by clinical significance and significant results of univariate analysis (including age; gender; BMI; smoking status; undergoing general anesthesia surgery; ventilator application; ICU experience; CCI total score; use of antibiotics, tranquilizers, and glucocorticosteroids; and other related factors). A multilevel model approach was considered to explore factors that were associated with the incidence of HAP, because of the nested structure of the data (i.e., patients within different departments and departments within hospitals). Thus, a multilevel regression analysis was performed to determine factors associated with the occurrence of HAP by using MLwin software, version 2.10 (15). The occurrence of HAP was treated as the dependent variable. A random effects model was used to analyze the association between risk factors and HAP at three levels simultaneously so that the contribution of individual-, department-, and hospital -level data to the occurrence of HAP was partitioned.

## Results

### Characteristics of All Patients and Incidence of HAP

A total of 7,324 patients were contacted. All of them had complete data and were included in this analysis. Baseline characteristics of all patients were shown in Table 1. Of all patients, males accounted for 50.9%. The mean age of all patients was 74.51 years (SD = 7.35), and 32.5% of patients had primary school qualification. Regarding the department, 21.93% of patients came from ICU, 28.06% from orthopedics, 19.18% from neurology, 7.62% from neurosurgery, 7.05% from general surgery, and 6.23% from cardiovascular. The average length of hospital stay was 16.39 days (SD = 16.18); 1,386 patients spent 15 days or more in hospital, of which 285 patients suffered from HAP, accounting for 50.35% of all HAP patients. The percentage of patients with a BMI of 18.5–23.9 was 50.44%. In addition, 78.22% of patients had no smoking experience and 36.78% underwent general anesthesia surgery. A total of 478 patients received ventilator support and 770 patients had a CCI score of >7. Most patients (86.85%) were free from disorders of consciousness; in addition, 13.79% used glucocorticosteroids, 63.49% used antibiotics, and 13.90% took tranquilizers.

**Table 1:** Baseline characteristics of older patients with hospital acquired pneumonia and without hospital acquired pneumonia (*n* = 7,324).

**Variables**	**Total** **(*n* = 7324)**	**Patient with hospital acquired pneumonia** **(*n* = 566)**	**Patient without hospital acquired pneumonia** **(*n* = 6,758)**	***p*-value**
Female	3,596 (49.10)	240 (42.40)	3,356 (49.66)	<0.001
Age in years	74.51 ± 7.35	76.35 ± 7.53	74.35 ± 7.32	<0.001
Age group				<0.001
65–69	2,362 (32.25)	130 (22.97)	2,232 (33.03)	
70–74	1,715 (23.42)	117 (20.67)	1,598 (23.65)	
75–79	1,365 (18.64)	114 (20.14)	1,251 (18.51)	
80–84	1,072 (14.64)	113 (19.96)	959 (14.19)	
≥85	810 (11.06)	92 (16.25)	718 (10.62)	
Education level				0.46
Illiteracy	1,791 (24.45)	151 (26.68)	1,640 (24.27)	
Primary school	2,380 (32.5)	174 (30.74)	2,206 (32.64)	
Middle school	1,460 (19.93)	118 (20.85)	1,342 (19.86)	
Senior high school	914 (12.48)	72 (12.72)	842 (12.46)	
College degree or above	779 (10.64)	51(9.01)	728 (10.77)	
Department				<0.001
ICU	1,606 (21.93)	271(47.88)	1,335 (19.75)	
Orthopedics	2,055 (28.06)	50 (8.83)	2,005 (29.67)	
Neurology	1,405 (19.18)	113 (1.54)	1,292 (17.64)	
Neurosurgery	558 (7.62)	52 (9.19)	506 (7.49)	
General surgery	516 (7.05)	12 (2.12)	504 (7.46)	
Cardiovascular	456 (6.23)	31 (5.48)	425 (6.29)	
Other surgery	443 (6.05)	18 (3.18)	425 (6.29)	
Other medicine	285 (3.89)	19 (3.36)	266 (3.94)	
Length of bedridden days				<0.001
1–3	2,202 (30.07)	31 (5.48)	2,171 (32.12)	
4–7	2,166 (29.57)	81 (14.31)	2,085 (30.85)	
8–14	1,570 (21.44)	169 (29.86)	1,401 (20.73)	
≥15	1,386 (18.92)	285 (50.35)	1,101 (16.29)	
BMI group (*n* = 7319)				0.003
18.5–23.9	3,692 (50.44)	295 (52.21)	3,397 (50.30)	
<18.5	628 (8.58)	67 (11.86)	561 (8.31)	
>23.9 and <28	2,341 (31.99)	167 (29.56)	2,174 (32.19)	
≥28	658 (8.99)	36 (6.37)	622 (9.21)	
Smoking				<0.001
Never smoking	5,729 (78.22)	398 (70.32)	5,331 (78.88)	
Smoking	1,105 (15.09)	109 (19.26)	996 (14.74)	
Ex-smokers	490 (6.69)	59 (10.42)	431 (6.38)	
Receiving general anesthesia operation (yes)	2,694 (36.78)	139 (24.56)	2,555 (37.81)	<0.001
Application of ventilator (yes)	478 (6.53)	108 (19.08)	370 (5.47)	<0.001
CCI total score	5 ± 1.86	5.6 ± 1.84	4.95 ± 1.86	<0.001
Disorder of consciousness (yes)	963 (13.15)	279 (49.29)	684 (10.12)	<0.001
Glucocorticosteroids use (yes)	1,010 (13.79)	177 (31.27)	833 (11.37)	<0.001
Antibiotics use (yes)	4,650 (63.49)	528 (93.29)	4,122 (60.99)	<0.001
Tranquilizers use	1,018 (13.9)	180 (31.80)	838 (12.40)	<0.001
Bed to nurse ratio (not attained)	3,315 (48.02)	3,071 (48.31)	244 (44.69)	0.104

*CCI, Charlson Comorbidity Index; ICU, Intensive care unit*.

**Figure 2:**
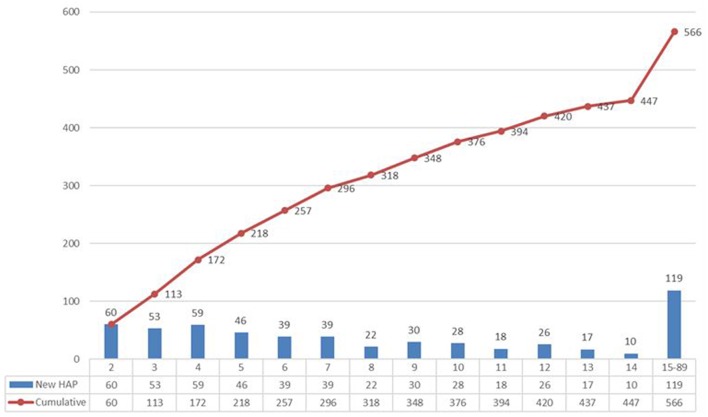
Daily and cumulative of occurrence of HAP in patients with HAP. The median of duration from admission to diagnosis of HAP was 7 days (IQR, 4–13) and the mode was 2 days.

Among all participating patients, 566 patients were diagnosed with HAP after they were admitted to hospital. The incidence density of HAP of all participants was 13.9 per 1,000 person-days and it varied among the different departments. The incidence density of HAP in the ICU was 30.1 per 1,000 person-days, which was the highest. Neurosurgery (18.7‰) and neurology medicine (16.6‰) also had higher incidence density than other departments (see **Table 3**). As shown in Figure 2, the median of duration from admission to diagnosis of HAP was 7 days (IQR, 4–13) and the mode was 2 days. We compared the demographic characteristics and clinical data between patients with HAP and patients without HAP, and found that there were statistical differences in gender, age, length of bedridden days, BMI, smoking, department, undergoing general anesthesia surgery, ventilator application, CCI score, disorders of consciousness, tranquilizer use, glucocorticosteroid use, and antibiotic use (all *p* < 0.05) (Table 1).

### Factors Associated With HAP

The above factors with inter-group differences were subsequently entered into the multilevel regression model. Because different nursing conditions may affect the occurrence of HAP, the bed -to -nurse ratio (attained or not attained) was considered in the model. The multilevel regression model included three levels: individual, department, and hospital. As shown in Table 2, there was no significant variance for HAP at the hospital level (0.332, *t* = 1.875, *p* > 0.05). There was significant variance for HAP among different departments (0.553, *t* = 4.320, *p* < 0.01). The patients from the ICU had higher risk for HAP than those from non-ICU departments (1.182, *t* = 6.031, *p* < 0.001). Patients over 75 years of age were at increased risk compared with those <70 years (*p* < 0.05). Patients with disorders of consciousness were at higher risk compared with those without disorders of consciousness (1.146, *t* = 8.815, *p* < 0.001). High CCI score, use of antibiotics, and use of glucocorticoids were related to the occurrence of HAP (all *p* < 0.05), while patients who received general anesthesia for surgical intervention had lower risk than those who did not (*p* < 0.01). There was no association between smoking, gender, BMI, length of bedridden days, bed-to-nurse ratio, ventilator application, and tranquilizer use and the occurrence of HAP (all *p* > 0.05).

**Table 2:** Multilevel regression analysis of factors associated with HAP.

**Variables**	**Estimation**	**Standard error**	***t* ratio**	***P*-value**	**Hospital level estimate (SE)**	**Department level estimate (SE)**
Constant	−3.867	0.334	−11.578	**<0.001**	0.332 (0.177)	0.553 (0.128)
Age					*T* = 1.875	*T* = 4.320
70–74	0.166	0.134	1.239	>0.05	*P* > 0.05	*P* < 0.01
75–79	0.308	0.143	2.154	**<0.05**		
80–84	0.425	0.150	2.833	**<0.05**		
85 or more	0.524	0.166	3.157	**<0.05**		
Male	0.098	0.107	0.916	>0.05		
Length of bedridden days	−0.000	0.000	0	>0.05		
**BMI**						
<18.5	0.174	0.157	1.108	>0.05		
24–27.9	0.022	0.106	0.207	>0.05		
≥28	−0.035	0.180	−0.194	>0.05		
Ex-smokers	0.069	0.137	0.504	>0.05		
Smoking	0.106	0.173	0.613	>0.05		
ICU	1.182	0.196	6.031	**<0.001**		
Receiving general anesthesia operation	−0.767	0.128	−5.992	**<0.01**		
Application of ventilator	0.259	0.159	1.629	>0.05		
Disorder of consciousness	1.146	0.130	8.815	**<0.001**		
Glucocorticosteroids use	0.619	0.122	5.073	**<0.01**		
Antibiotics use	2.211	0.180	12,283	**<0.001**		
Tranquilizers use	0.060	0.136	0.441	>0.05		
CCI total score	0.057	0.026	2.192	**<0.05**		
Bed to nurse ratio (attained)	0.152	0.159	0.956	>0.05		
Total variance explained	Individual level 81.98%				6.13%	11.88%

*CCI, Charlson Comorbidity Index; ICU, Intensive care unit; BMI, body mass index*.

These individual level factors contributed to the occurrence of HAP and explained 81.98% of variance.

### Distribution of Factors Associated With HAP Among Different Departments

In the multilevel analysis, the hospital contributed to 6.13% of the variance, but its contribution to the occurrence of HAP was not significant. The department explained 11.88% of the variance and significantly contributed to the occurrence of HAP (see Table 2). Because of the significant variance of HAP explained by the department-level results, we further analyzed the distribution of factors associated with HAP in patients with HAP among different departments. As shown in Table 3, there were significant differences in the distribution of age, disorders of consciousness, CCI total score, undergoing general anesthesia surgery, use of glucocorticoids, and use of antibiotics in patients with HAP among different departments (all *p* < 0.001). The results showed that 59.5% of disorders of consciousness were in HAP patients in the ICU department, 18.3% in HAP patients in the neurology medicine department, and 14.0% in HAP patients in the neurosurgery department. The use of glucocorticoids was higher in the ICU (54.2%) and neurosurgery (14.1%) departments compared with other departments. The use of antibiotics was relatively high in the ICU (49.8%), neurology medicine (18.0%), neurosurgery (9.1%), and orthopedics (8.5%) departments. Most patients with HAP who received general anesthesia for surgical intervention came from the ICU (50.4%); 16.5% came from the orthopedics department and 15.8% from the neurosurgery department. Higher CCI total score was found in the ICU (5.7 ± 2.1), neurology medicine (5.3 ± 1.3), general surgery (5.8 ± 2.7), cardiovascular (5.5 ± 1.5), other surgery (5.4 ± 2.4), and other medicine (5.1 ± 1.7) departments. Older age was found in the orthopedics, neurology medicine, general surgery, other medicine, and ICU departments.

**Table 3:** The incidence density of HAP among different departments and distribution of related factors of HAP in patients with HAP among different departments.

**Variables**	**ICU**	**Orthopedics**	**Neurology medicine**	**Neurosurgery**	**General surgery**	**Cardiovascular**	**Other surgery**	**Other medicine**	**χ^2^/*F***	***p***
Disorder of consciousness	166 (59.4%)	9 (3.2%)	51 (18.3%)	39 (14.0%)	3 (1.1%)	6 (2.2%)	3 (1.1%)	2 (0.7%)	82.67	<0.001
Glucocorticosteroids use	96 (54.2%)	17 (9.6%)	14 (7.9%)	25 (14.1%)	3 (1.7%)	5 (2.8%)	9 (5.1%)	8 (4.6%)	35.43	<0.001
Antibiotics use	263 (49.8%)	45 (8.5%)	95 (18.0%)	48 (9.1%)	12 (2.3%)	28 (5.3%)	18 (3.4%)	19 (3.6%)	26.35	<0.001
CCI total score	5.7 ± 2.1	4.1 ± 1.2	5.3 ± 1.3	4.3 ± 1.3	5.8 ± 2.7	5.5 ± 1.5	5.4 ± 2.4	5.1 ± 1.7	164.41	<0.001
Age in years	75.6 ± 7.5	79.9 ± 7.5	77.5 ± 6.9	73.9 ± 6.8	79.9 ± 7.1	69.5 ± 4.2	76.8 ± 8.7	76.3 ± 7.5	6.98	<0.001
Incidence density Mean (SD)	30.1‰ (0.087)	4.0‰ (0.034)	16.6‰ (0.069)	18.7‰ (0.073)	2.1‰ (0.018)	10.8‰ (0.050)	3.3‰ (0.018)	13.1‰ (0.058)	Total incidence density 13.9‰	

*CCI, Charlson Comorbidity Index; ICU, Intensive care unit*.

## Discussion

HAP is one of the leading hospital-acquired infections worldwide and is associated with elevated morbidity, mortality and increased hospital costs (1). Few studies have been conducted on the epidemiology of HAP among older bedridden patients when the incidence of HAP in the older people increases with age (16). This study aimed to identify the incidence and related factors of HAP among older bedridden patients by using representative departments in 25 hospitals with different administrative levels in six provinces and cites in China. Our study demonstrates that the incidence density of HAP among older bedridden patients is as high as 13.9% person-days; this is consistent with the results from other countries that the incidence of HAP is between 8 and 10% of patients admitted to hospital for the older people units (16, 17). There was no significant variance for HAP at the hospital level in this study. Thus, the current result is the first time to demonstrate the incidence of HAP among older bedridden patients in China. The incidence of HAP varied among different departments. In line with previous studies, we found that the incidence rate of HAP was highest in the ICU among the selected departments in this study (7), followed by the departments of neurosurgery and neurology.

Our study found that age, disturbance of consciousness, total CCI score, ICU admission, glucocorticoid use, and antibiotic use were associated with occurrence of bedridden related HAP. Previous studies have also shown that older age and unconsciousness are associated with HAP (9, 18, 19). Older bedridden patients often have complicated illnesses and have to accept multiple types of medicines, such as glucocorticoids, and antibiotics. It is possible that long-term use of glucocorticoids reduces the body's immune function and inhibits neutrophil chemotaxis (1, 20). In this study, most patients (437/566) had antibiotic use before diagnosing HAP. This is consistent with other studies showing that antibiotic use is a common risk factor for HAP (1, 21). The improper use of antibiotics has resulted in the imbalance of bacterial flora and the emergence of multi-drug-resistant strains, especially in hospitals, which has increased the incidence of nosocomial infections (22). Proper use of antibiotics can help to decrease the infection rate of HAP. There has been new progress in the use of antibiotics. New evidence-based guidelines for the diagnosis and treatment of these entities were released by the Infectious Diseases Society of America and the American Thoracic Society in 2016, which sets 7 days as the recommended duration of antibiotic therapy, regardless of the isolated pathogen(s). The available data suggest there was no difference between short-course (7–8 days) and long-course (10–15 days) antibiotic regimens in regard to mortality (8). The reduction of the antibiotic use will reduce days of exposure to antibiotics, minimizing a risk of developing resistance in bedridden patients (23). These findings suggest that antibiotics and glucocorticoids should be used cautiously during the treatment.

The Charlson comorbidity index, a method of predicting mortality by classifying or weighting comorbid conditions (comorbidities), has been widely utilized to measure disease burden, severity and prognosis since it was published in 1987 (24). Comorbidities were considered as an important predictor of mortality of pneumonia in older patients as the more comorbidities the patients have, the more susceptible they are to have infection (25–28). Our study also found higher CCI total score was associated with the occurrence of HAP. Thus, a comprehensive assessment of complications should be made at the beginning of hospitalization for older patients to prevent HAP.

It is noteworthy that we found that patients receiving general anesthesia for surgical intervention had a decreased risk of HAP. This finding may be explained by the fact that general anesthesia requires patients to have good cardiopulmonary function and be able to withstand general anesthesia. Therefore, the physical condition of these patients may be better than that of patients who cannot undergo general anesthesia, and they are less likely to suffer pneumonia infection because of their suitability for more comprehensive treatment and care, such as sputum aspiration after completion of general anesthesia surgery (29). The reasonable and effective treatment of primary diseases and rapid recovery from the disease may be the key factors to prevent nosocomial infection. Further research is needed to demonstrate the association between general anesthesia surgery and the occurrence of HAP.

In this study, 47.88% of patients with HAP had ICU experience, and the multilevel regression analysis indicated that the ICU department was significantly associated with the occurrence of HAP. It was reported that HAP is the most common infection acquired in the ICU (9). Previous research showed that patients who had been in the ICU for 3–4 days were three times more likely to be infected than those with a stay of 1–2 days, and those who had been in the ICU for 21 days or more were at 33 times the risk of patients with a hospital stay of 1–2 days (30). ICU patients usually have more serious illness and accept more invasive operations, such as nasal feeding, ventilation, and these operations may increase infection risk to patients (31). This study also found that patients in the ICU had the most HAP-related factors, including higher age and CCI total score, disturbance of consciousness, and use of glucocorticoids and antibiotics, which might also explain why the incidence of HAP in the ICU was the highest. We also found higher incidence of HAP in the department of neurosurgery and neurology medicine. Most patients in theses departments had severe diseases, such as stroke, epilepsy, brain tumor, brain injury and cerebral hemorrhage, which may also have high CCI total score, disturbance of consciousness, and the use of glucocorticoids and antibiotics. These factors were found to be significantly related to HAP. Current findings suggest we should also focus on the prevention of HAP in the department of neurosurgery and neurology medicine while we prevent the occurrence of HAP in ICU.

The current study found differences in gender, BMI, smoking, length of bedridden days, ventilator application, and use of tranquilizers between patients with HAP and patients without HAP. Several previous studies demonstrated that BMI, smoking, ventilator application, and use of tranquilizers were associated with the occurrence of HAP (32–36). However, our study did not find a correlation between these factors and HAP. The inconsistent results may be due to differences in the sampling method and subjects included in this study. The results of this study can only represent the situation of older bedridden patients in China.

This study investigated the incidence and related factors of HAP among older bedridden patients in China. This study included older bedridden patients from different types of department of 25 hospitals in only six provinces and municipalities, a more representative sample from other provinces is needed to generalize the results to other regions in China in future studies. Furthermore, we excluded patients who had more than one type of MCI at the time of enrollment. Thus, the incidence of HAP may be underestimated.

## Conclusion

In conclusion, the incidence of HAP among older bedridden patients is as high as 13.9 per 1,000 person days in this study. The incidence density of HAP varies among different departments. In addition to the ICU, the department of neurosurgery and neurology medicine have relatively high incidence of HAP. Several factors including age, disturbance of consciousness, ICU admission, CCI total score, antibiotic use, and glucocorticoid use are associated with HAP. Current results suggest that effective evaluation and management of these related factors of HAP in older bedridden patients should be intensified to reduce the occurrence of HAP.

## Data Availability

All datasets generated for this study are included in the manuscript/supplementary files.

## Author Contributions

XW designed the study. JS designed the statistical methodology, analyzed the data, reviewed, and edited the manuscript. ZL conducted data analysis. JJia and XY wrote the manuscript. YZ, JC, FL, YiL, GL, BS, JJin, Yi-lL, XW, SC, and LY collected the data. All authors critically reviewed and proofed the manuscript.

### Conflict of Interest Statement

The authors declare that the research was conducted in the absence of any commercial or financial relationships that could be construed as a potential conflict of interest. The handling editor declared a shared affiliation, though no other collaboration, with one of the authors JJin.
